# Intravenous magnesium prevents atrial fibrillation after coronary artery bypass grafting: a meta-analysis of 7 double-blind, placebo-controlled, randomized clinical trials

**DOI:** 10.1186/1745-6215-13-41

**Published:** 2012-04-20

**Authors:** Wan-Jie Gu, Zhen-Jie Wu, Peng-Fei Wang, Lynn Htet Htet Aung, Rui-Xing Yin

**Affiliations:** 1 Department of Cardiology, Institute of Cardiovascular Diseases, the First Affiliated Hospital, Guangxi Medical University, 22 Shuangyong Road, Nanning 530021, Guangxi, People's Republic of China; 2 Department of Colorectal and Anal Surgery, The First Affiliated Hospital, Guangxi Medical University, Nanning, Guangxi, People's Republic of China; 3 Department of Orthopaedics, China-Japan Union Hospital, Jilin University, Changchun, Jilin, People's Republic of China

## Abstract

**Background:**

Postoperative atrial fibrillation (POAF) is the most common complication after coronary artery bypass grafting (CABG). The preventive effect of magnesium on POAF is not well known. This meta-analysis was undertaken to assess the efficacy of intravenous magnesium on the prevention of POAF after CABG.

**Methods:**

Eligible studies were identified from electronic databases (Medline, Embase, and the Cochrane Library). The primary outcome measure was the incidence of POAF. The meta-analysis was performed with the fixed-effect model or random-effect model according to heterogeneity.

**Results:**

Seven double-blind, placebo-controlled, randomized clinical trials met the inclusion criteria including 1,028 participants. The pooled results showed that intravenous magnesium reduced the incidence of POAF by 36% (RR 0.64; 95% confidence interval (CI) 0.50-0.83; *P *= 0.001; with no heterogeneity between trials (heterogeneity *P *= 0.8, *I*^2 ^= 0%)).

**Conclusions:**

This meta-analysis indicates that intravenous magnesium significantly reduces the incidence of POAF after CABG. This finding encourages the use of intravenous magnesium as an alternative to prevent POAF after CABG. But more high quality randomized clinical trials are still need to confirm the safety.

## Introduction

Postoperative atrial fibrillation (POAF) is the most common complication encountered following coronary artery bypass grafting (CABG). It generally occurs between 24 and 96 h postoperatively, with a peak incidence on the second postoperative day [1-3]. With continuous electrocardiographic monitoring, the incidence of atrial fibrillation after CABG reported in previous studies varies from 10% to 50% [1,2,4], and this incidence has not decreased despite improvements in anesthetic and surgical techniques [5,6]. Furthermore, atrial fibrillation potentially leads to complications, including stroke [7-9], extended duration of hospitalization [7-10], and increasing costs [1,9,10].

The etiology of atrial fibrillation after CABG is unclear. The cause may be multifactorial, such as advanced age, previous history of atrial fibrillation, and low blood magnesium concentrations [11-13]. There are many pharmacologic agents to prevent POAF, but none of them are effective for all patients and are free of complications [14]. Particularly, magnesium seems to be with great promise to prevent POAF following CABG.

A previous meta-analysis of magnesium for prevention of atrial fibrillation after CABG including eight randomized controlled trials was published in 2005 [15]. The analysis showed that intravenous magnesium is associated with a significant reduction in the incidence of atrial fibrillation after CABG, with a relative risk of 0.64 (95% confidence interval (CI) 0.47-0.87). But this meta-analysis included some clinical studies which had a modest sample size. Moreover, some of these included studies are of low quality. Recently, an increasing number of studies on the efficacy of intravenous magnesium on the prevention of POAF have been published. These studies have contrasted in these randomized controlled trials. Therefore, we performed an updated meta-analysis only based on double-blind, placebo-controlled, randomized clinical trials to re-examine the effects of intravenous magnesium on the prevention of POAF after CABG.

## Materials and methods

### Search strategy and selection criteria

Two investigators (WJG and ZJW) independently searched the literatures collected in Medline, Embase, and the Cochrane Library up to August 1, 2011. Search terms included: magnesium, fibrillation. The searches were limited to English publications in humans. We screened the reference lists of included studies and related publications. The results were then hand searched for eligible trials. We did not include abstracts or meeting proceedings. This search strategy was performed iteratively until no new potential citations could be found on review of the reference lists of retrieved articles.

We included full-text publications when the following inclusion criteria were met: adult patients undergoing CABG only; randomized allocation to magnesium group or control group (only placebo); double-blind, placebo-controlled, randomized clinical trial; and providing available data on the incidence of POAF. Exclusion criteria included: (a) unavailable duration of follow-up; and (b) some patients reported with pre-existing atrial fibrillation. The trials with small sample size of (*n *< 10) were also excluded to avoid selection bias.

### Data extraction and quality assessment

Two investigators (WJG and ZJW) independently extracted the following information from each study: the first author's name, year of publication, country of origin, participants' characteristics, study design (randomized), type of controls (placebo), data collection (prospective or not), sampling method (consecutive or not), type of blinding (double blind), duration of follow-up, regimen of magnesium administration, the timing of magnesium infusion was initiated (preoperative, intraoperative, or postoperative), number, mean age and percentage of males in each trial, total number of individuals, and the incidence of POAF in each group. When the same population was reported in several publications, we retained only the most informative article or completed study to avoid duplication of information. Any disagreements were resolved through discussion and consensus.

The methodological quality of the studies included in the meta-analysis was scored using validated Jadad 5-point scale. The scale consists of three items describing randomization (0-2 points), double-blind (0-2 points), and drop-outs and withdrawals (0-1 points) in the report of a randomized controlled trial. One point was given when one quality criterion was met. The quality scale ranges from 0 to 5 points. Higher scores indicate better reporting. The studies are said to be of low quality if the Jadad score is ≤ 2 and high quality if the score is ≥ 3 [16,17].

### Statistical analysis

Data were analyzed using Stata version 11 (Stata Corporation, College Station, TX, USA). A statistical test with a *P *value less than 0.05 was considered significant. The incidence of POAF was treated as dichotomous variables and was expressed as risk ratio (RR) with 95% CI for each study. Pooled estimates of efficacy were calculated using the Man-tel-Haenszel fixed-effects model [18]. But if there was heterogeneity, the following methods were used to deal with it: (a) subgroup analysis; (b) sensitivity analysis performed by excluding trials which potentially biased the results. If heterogeneity still potentially existed, the DerSimonian and Lair random-effects model was used. A test for heterogeneity, defined as variation among the results of individual trials for a given treatment beyond that expected from chance, was used to assess whether the magnitude of a given preventive effect varied between the trials. We assessed heterogeneity with *I*^2^, which describes the percentage of total variation across studies due to heterogeneity rather than chance. *I^2 ^*can be calculated as: *I*^2 ^= 100% × (Q-df)/Q(Q = Cochrane's heterogeneity statistics, df = degrees of freedom). Negative values of *I^2 ^*equaled zero, so that *I*^2 ^ranged between 0% (that is no observed heterogeneity) and 100%. High values would show increasing heterogeneity. Studies with an *I^2 ^*statistic of 25% to 50% are considered to have low heterogeneity, those with an *I^2 ^*statistic of 50% to 75% are considered to have moderate heterogeneity, and those with an *I^2 ^*statistic of > 75% are considered to have a high degree of heterogeneity [19]. The presence of publication bias was evaluated by using the Egger test [20].

## Results

**Figure 1:**
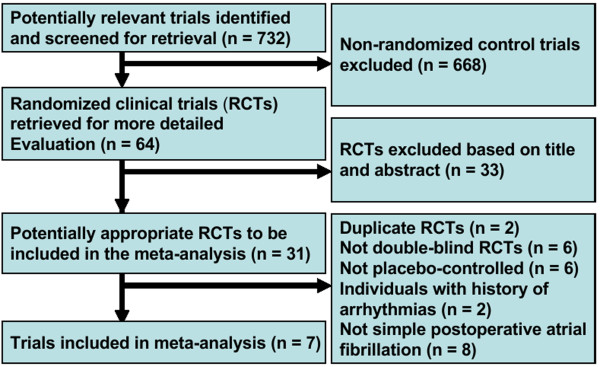
**Process of study selection of double-blind, placebo-controlled, randomized trials**.

Seven double-blind, placebo-controlled, randomized clinical trials consisting of 1,028 individuals were included in this study. Four of the eight randomized controlled trials published by Alghamdi *et al*. [15] were also included and the remaining four trials were excluded because they were non-double-blind. Three eligible studies were published after 2003. The flow of identified studies through the selection process is shown in Figure 1.

### Description of eligible trials

The efficacy of intravenous magnesium with placebo on the prevention of POAF was compared in these trials. The baseline characteristics of included studies are shown in Table 1 and the design characteristics are presented in Table 2. Of the seven trials, two were done in the USA, one in UK, one in Turkey, one in Switzerland, one in Iran, and one in Pakistan. The number of participants ranged from 50 to 345. All trials included both men and women. The total dosage of intravenous magnesium in the intervention groups ranged from 8 to 100 mmol (one trial [21] was not available). The follow-up time ranged from 1 to 5 days (one trial [11] was followed up until atrial fibrillation developed and needed therapeutic intervention). All trials reported perioperative prophylactic use of intravenous magnesium: one trial [21] was initiated during the preoperative period, two trials [11,22] during the intraoperative period, and four trials [23-26] during the postoperative period.

**Table 1:** Characteristics of studies included in the meta-analysis

Author	*n*	Regimen of magnesium administration^a^	Control regimen (route)	Total dose Mg^2+ ^(mmol)	Magnesium		Controls		POAF	
					Age (years), (male, %)	*n*	Age (years), (male, %)	*n*	Magnesium (%) Controls (%)	
Fanning *et al.*[23]	99	168 mEq over the first 4 postoperative days	5% dextrose solution (IV)	84 mmol	59 (43-75)	49 (71.4)	62 (42-79)	50 (78)	7/49 (14.3)	14/50 (28)
Colquhoun *et al.*[24]	130	50 mmol during the first 48 h after surgery	5% dextrose solution (IV)	50 mmol	57.1 ± 8.4	66 (83.3)	58.7 ± 7.9	64 (79.7)	11/66 (16.7)	15/64 (23.4)
Nurozler *et al.*[26]	50	100 mEq on the first operative day and 25 mEq per day from second to fifth days	Placebo (IV)	100 mmol	56.3 ± 1.6	25 (92)	53.6 ± 2.0	25 (92)	1/25 (4)	5/25 (20)
Treggiari-Venzi *et al.*[25]	98	4 g per 24 h continuous infusion for 72 h starting within 1 h of arrival in the ICU	0.9% NaCl solution (IV)	48 mmol	65 (46-81)	47 (89.4)	65 (37-88)	51 (84.3)	11/47 (23)	14/51 (27)
Hazelrigg *et al.*[21]	202	80 mg/kg (ideal body weight) before cardiopulmonary bypass (CPB), 8 mg/kg/h (ideal body weight) intravenous (IV) infusion continued for 48 h	5% dextrose solution (IV)	NA	62.1 ± 9.5	105 (74)	63.7 ± 11.1	97 (68)	32/105 (30.5)	41/97 (42.3)
Najafi *et al.*[11]	345	2 g after induction of anesthesia until the start of cardiopulmonary bypass (CPB) and 8 g after CABG until 24 h after surgery	Placebo (IV)	40 mmol	59.1 (9.1)	166 (75.9)	59.7 (9.9)	179 (76.5)	12/166 (7.2)	22/179 (12.3)
Hamid *et al.*[22]	104	2 g after intubation	0.9% NaCl solution (IV)	8 mmol	58.3 ± 7.6	53 (98)	56.3 ± 8.9	51 (86)	2/53 (3.77)	5/51 (9.8)

^a^To convert units of g and mEq to mmol, the following conversions were used; 1 g = 4 mmol = 8 mEq for MgSO4.

CABG, coronary artery bypass grafting; CPB, cardiopulmonary bypass; ICU, intensive care unit;IV, intravenous; *n*, number of participants; NA, data not available; POAF, postoperative atrial fibrillation;

**Table 2:** Included studies design characteristics

Author	Publication year	Country	Study design	Data collection	Sampling method	Blind	Follow-up (days)	Jadad score
Fanning *et al.*[23]	1991	USA	Randomized, placebo-controlled	Prospective	Consecutive	DB	4	4
Colquhoun *et al.*[24]	1993	UK	Randomized, placebo-controlled	Prospective	Consecutive	DB	4	4
Nurozler *et al.*[26]	1996	Turkey	Randomized, placebo-controlled	Prospective	Consecutive	DB	5	3
Treggiari-Venzi *et al.*[25]	2000	Switzerland	Randomized, placebo-controlled	NA	NA	DB	3	5
Hazelrigg *et al.*[21]	2004	USA	Randomized, placebo-controlled	Prospective	NA	DB	5	4
Najafi *et al.*[11]	2007	Iran	Randomized, placebo-controlled	Prospective	Consecutive	DB	Until AF developed and needed therapeutic intervention	4
Hamid *et al.*[22]	2008	Pakistan	Randomized, placebo-controlled	NA	NA	DB	1	4

AF, atrial fibrillation; DB, double-blind; NA, data not available

### Quality assessment of the trials

The trials included in this meta-analysis appeared to have been reasonably designed and conducted. All studies had a statement regarding randomization and double-blind. Four trials described the methods of randomization. Four trials reported the withdrawals or dropouts. All trials described the main outcome, and no missing data seemed to influence the results. The quality of the included studies was assessed by the Jadad score. The median Jadad score of the studies included was 4 (range from 3 to 5, Table 2).

### The incidence of POAF

Analysis of pooled prevalence of preoperative patient group characteristics revealed that no differences were observed for history of coexistence of basic diseases (for example diabetes mellitus, hypertension) and routine prophylactic therapies (for example β-blockers; Table 3).

**Table 3:** Perioperative variables of the patients

Variable	Magnesium (% (*n*))	Control (% (*n*))	*x*^2 ^value	*P *value	Total prevalence (% (*n*))
Diabetes mellitus [11,24-26]	22.4 (68/304)	21.9 (70/319)	0.016	0.9	22.2 (138/623)
Hypertension [24-26]	31.2 (43/138)	27.1 (38/140)	0.543	0.5	29.1 (81/278)
β-blocker [21-25]	45.6 (146/320)	48.6 (152/313)	0.548	0.5	47.1 (298/633)

**Figure 2:**
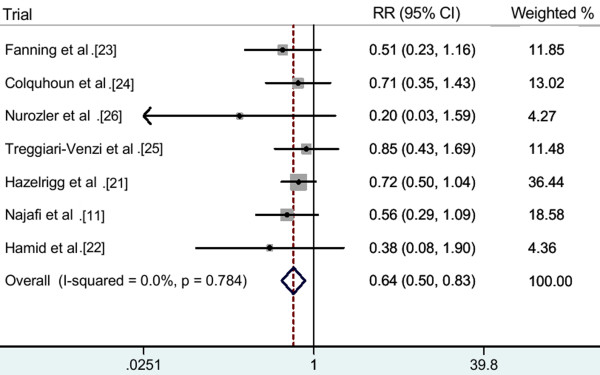
**All included studies, RR (fixed effect model)**.

Pooling all seven trials, of 511 patients in the pooled intervention (intravenous magnesium) group, 76 developed POAF, compared to 116 out of 517 patients in the pooled control group. The pooled analysis showed that intravenous magnesium significantly reduced the incidence of POAF by 36% (RR 0.64; 95% CI 0.50-0.83; *P *= 0.001; Figure 2), with no heterogeneity between trials (heterogeneity *P *= 0.8; *I*^2 ^= 0%).

Subgroup analyses were done according to data collection, sampling method and the duration of follow-up. Pooled results of five prospective trials [11,21,23,24,26] showed intravenous magnesium significantly reduced the incidence of POAF by 37% (RR 0.63, 95% CI 0.48-0.83; *P *= 0.001; heterogeneity *P *= 0.7, *I*^2 ^= 0%). Pooled results of four consecutive patients trials [11,23,24,26] showed that intravenous magnesium significantly reduced the incidence of POAF by 44% (RR 0.56, 95% CI 0.37-0.83; *P *= 0.005; heterogeneity *P *= 0.7, *I*^2 ^= 0%). Exclusion of the Hamid *et al*. trial [22] in which the duration of follow-up is just 1 day yielded similar results (RR 0.66, 95% CI 0.51-0.85; *P *= 0.002; heterogeneity *P *= 0.8; *I*^2 ^= 0%). The summary of subgroup analyses results is shown in Table 4.

**Table 4:** The summary of subgroup analyses results

Subgroup analysis	Studies (*n*)	Participants (*n*)	RR (95% CI)	*I*^2^(%)	*P *heterogeneity	*P *value
Prospective	5	826	0.63 (0.48-0.83)	0.00	0.7	0.001
Consecutive	4	624	0.56 (0.37-0.83)	0.00	0.7	0.005
Follow-up	6	924	0.66 (0.51-0.85)	0.00	0.8	0.002

CI, confidence interval; *n*, number of studies; RR, risk ratio

### Publication bias

**Figure 3:**
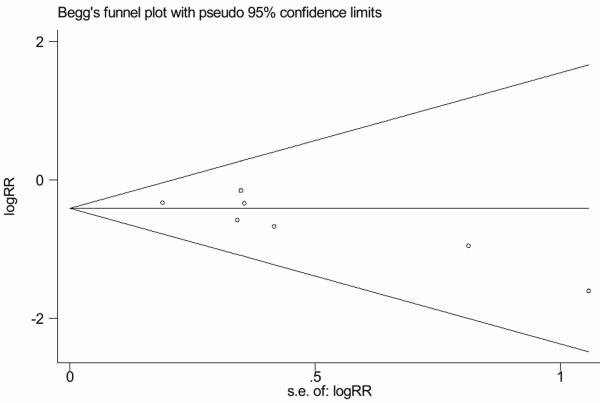
**Tests for publication bias for RR of the incidence of POAF**.

Assessment of publication bias using Egger's test showed that moderate publication bias existed among the included trials (Egger's test: *P *= 0.045; Figure 3).

## Discussion

This is a further meta-analysis to evaluate the relationship between intravenous magnesium and POAF. All trials included in this analysis are double-blind, placebo-controlled, randomized clinical trials. The data can give greater power to assess the efficacy of intravenous magnesium on the prevention of atrial fibrillation after CABG. We combined the effect sizes of all seven included trials that used intravenous magnesium for preventing POAF through a fixed-effects model and found that intravenous magnesium significantly reduced the incidence of POAF by 36%. A meta-analysis of data collected before December 2003 [15] found a cumulative RR of 0.64 (95% CI: 0.47-0.87) for the randomized controlled trials. Our findings are consistent with this previous meta-analysis.

This meta-analysis shows diversity in the dosing, timing and duration of magnesium administration. The diversity accounts for the inconsistency in the reported outcomes of the included trials as listed in Table 1. In three [23,24,26] of the prospectively controlled clinical trials [11,21,23,24,26], intravenous magnesium significantly reduced the incidence of POAF after CABG. In three trials, magnesium was dosed for at least 2 consecutive days postoperatively. Given that the onset of POAF following CABG generally occurs between 24 and 96 h postoperatively, with a peak incidence on the second postoperative day and that it is often associated with hypomagnesaemia, intravenous magnesium supplementation during this period may play a key role in the suppression of POAF.

Demographic bias owing to generating the sequence of randomization inadequately may be another reason for the discordance in the reported results of magnesium prophylaxis. The biased variable, if it happens to be a powerful predictor of POAF, would apparently have a strong influence on the outcome of the study. For example, one trial [22] showed that prophylactic magnesium supplementation does not significantly reduce the incidence of POAF, patients in the magnesium group had a higher ratio of male gender (98% versus 86%, *P *= 0.02). This characteristic, male gender has been consistently a risk factor for the development of POAF.

Up to now, various preventive methods including pharmacologic and non-pharmacologic interventions have been proposed in the preventive strategy of POAF. Current evidences from meta-analyses [27-29] suggest that beta-blockers are effective and safe for most patients and advise that clinicians should not discontinue beta-blockers before cardiac surgery, unless contraindicated. Amiodarone can be safely added in patients at high risk for atrial fibrillation. In a recent meta-analysis, however, Patel *et al*. [30] found that amiodarone increases the risk of bradycardia and hypotension, particularly when administered intravenously. Meta-analyses of the clinical trials [8,29,31] investigating the effect of prophylactic pacing have consistently suggested that single- or dual-site atrial pacing significantly reduces the incidence of POAF; however, it is limited in practical use because of its complexity. Furthermore, there are some other pharmaceuticals such as statins [32,33], N-3 polyunsaturated fatty acids [34], and anti-inflammatory agents [35,36] being used to prevent POAF following CABG. However, the number of enrolled patients in these trails was small, and the pharmaceutical doses and administration times varied widely among studies. Thus, further studies are still necessary before confirmed conclusion. In a prospective, randomized, double-blind, placebo-controlled study, Cagli *et al*. [37] have concluded that low-dose amiodarone and magnesium combination is an effective, simple, well-tolerated, and possibly cost-effective regimen to prevent atrial fibrillation after CABG for high-risk patients. Perhaps appropriate combinations of these pharmacologic and non-pharmacologic interventions might be of benefit for further reducing POAF. In this meta-analysis, the patient population enrolled was quite homogeneous in its presentation. The studies included are of high quality, and all seven studies are double-blind, placebo-controlled, randomized trials having a Jadad score of ≥ 3. We combined all the studies using a fixed-effects mode and tested heterogeneity between trials with *I*^2 ^(0.0%) and with *P *value (0.8), indicating no heterogeneity.

Several potential limitations of this meta-analysis merit consideration. First, we accept that our meta-analysis included some clinical studies which had a modest sample size. Although we aimed to retrieve additional data from investigators, it was inevitable that some missing and unpublished data may still exist. Second, the exclusion of non-English-language studies and studies with fewer than 10 patients may lead to bias in effect size. In addition, follow-up time varied among included studies, and different total dose of intravenous magnesium was adopted in these studies. The discrepancy may explain clinical heterogeneity among studies, although no statistical heterogeneity is found.

## Conclusion

This meta-analysis of all seven double-blind, placebo-controlled, randomized clinical trials shows that intravenous magnesium significantly reduced the incidence of POAF after CABG by 36%. Pooled analysis of five prospective trials shows intravenous magnesium significantly reduced the incidence of POAF by 37%, and pooled analysis of four consecutive patient trials shows that intravenous magnesium significantly reduced the incidence of POAF by 44%. This finding encourages the use of intravenous magnesium as an alternative to prevent POAF after CABG but more high quality randomized clinical trials are still need to confirm the safety.

## Competing interests

The authors declare that they have no competing interests.

## Authors' contributions

WJG conceived the study, participated in the design, collected the data, and drafted the manuscript. ZJW collected the data, and performed statistical analyses. PFW and LHHA helped to collect the data. RXY conceived the study, participated in the design, and helped to draft the manuscript. All authors read and approved the final manuscript.
